# Failed induction of labor and associated factors among women undergoing induction at University of Gondar Specialized Hospital, Northwest Ethiopia

**DOI:** 10.1186/s12884-022-04476-7

**Published:** 2022-03-03

**Authors:** Tsion Tadesse, Nega Assefa, Hirbo Shore Roba, Yohannes Baye

**Affiliations:** 1 School of Midwifery, College of Medicine and Health Sciences, University of Gondar, P.O.Box: 196, Gondar, Ethiopia; 2 Department of Public Health, College of Health and Medical Sciences, Haramaya University, Harar, Ethiopia; 3 Department of Neonatal and Pediatric Nursing, College of Health and Medical Sciences, Haramaya University, Harar, Ethiopia

**Keywords:** Failed induction, Labor, Women, University of Gondar Specialized Hospital

## Abstract

**Background:**

Induction of labor is a process of artificially initiating labor to attain vaginal birth. Despite its vital role in the reduction of maternal mortality, the failure rate of induction and its contributing factors were not well studied in Ethiopia; particularly there was a limited study in the study area. This study aimed to assess the prevalence and factors associated with failed induction of labor among women undergoing induction at University of Gondar Specialized Hospital, Northwest Ethiopia.

**Methods:**

An institution-based retrospective cross-sectional study was conducted among 743 women undergoing induction at University of Gondar Specialized Hospital. A systematic random sampling method was used to draw a sample and the data were retrieved from the maternity registration books and medical records. Data were cleaned and entered into EpiData version 3.1 and SPSS version 20 used for analysis. Frequencies, proportions, and summary statistics were used to describe the study population and a multivariable logistic regression model was fitted to identify factors contributing to failed induction of labor. Odds ratio with 95% confidence interval computed and level of significance declared at *P*-value< 5%.

**Results:**

The prevalence of failed induction of labor was 24.4% (95% CI: 21.4, 27.9). Age ≤ 30 years (AOR = 3.7, 95% CI: 2.2,6.2), rural residence (AOR = 3.7, 95% CI: 2.4,5.8), being nulliparous (AOR = 2.1, 95% CI: 1.2,3.7), 5 or less Bishop Score (AOR = 3.4, 95% CI: 2.2,5.4), premature rupture of membrane (AOR = 2.7, 95% CI: 1.5,4.6), having pregnancy-induced hypertension (AOR = 4.0, 95% CI: 2.3,7.1), and artificial rupture of membrane with oxytocin (AOR = 0.2, 95% CI: 0.1, 0.4) were associated with failed induction of labor.

**Conclusions:**

One-fourth of women undergoing induction at University of Gondar Specialized Hospital had failed induction of labor. Age, residence, parity, bishop score, premature-rupture of the membrane, pregnancy-induced hypertension, and method of induction were independent predictors for failed induction of labor. The combination method of ARM with oxytocin, early detection and treatment of pregnancy-induced hypertension and premature rupture of the membrane are highly recommended for reducing failed induction of labor.

**Supplementary Information:**

The online version contains supplementary material available at 10.1186/s12884-022-04476-7.

## Background

Induction of labor (IOL) is an artificial stimulation of uterine contraction at 28 or more weeks of gestation but before spontaneous onset of labor to achieve vaginal delivery and it is a common practice in current obstetrics [1, 2]. IOL is a life-saving obstetrical intervention indicated only when the benefits of discontinuing the pregnancy outweigh the risks of continuation [3, 4]. The failure or success of induction may depend on the choice of induction methods, such as the pharmacological, mechanical, or a combination of both. Oxytocin and Misoprostol from the pharmacologic; ARM and Balloon catheter from the mechanical method are among the commonly used methods of labor induction [1, 5].

While there is a well-accepted definition of IOL, the definition of a failed induction of labor (FIOL) is less certain [6–8]. Nevertheless, a variety of criteria such as mode of delivery (vaginal versus cesarean) and certain time intervals within which active phase of labor achieved or adequate number of uterine contractions achieved are among the suggested criteria for diagnosing FIOL [1, 6]. The Federal Ministry of Health of Ethiopia (FMoH), defined FIOL as an inability to achieve adequate uterine contractions (3-5C/10 min/≥40s) after 6 to 8 h of oxytocin infusion with the use of its maximum dose [9]. Most other studies however defined FIOL as an inability to achieve vaginal delivery or birth through cesarean section (CS) [10–12].

Globally, IOL has been practiced in more than 20% of all pregnancies [2, 13, 14], and 20% of these pregnancies end up in delivery by CS [4]. In developed countries, up to 25% of the deliveries involve IOL and in developing countries in some settings, it was as high as those observed in the developed world ranging from 1.4% in Nigeria to 35.5% in Sri Lanka [15].

In Ethiopia, the national rate of failure of labor induction is unknown, but in some settings, it was reported as 17.3% in Hawassa, 21.4% in Jimma, and 19.7% in Dessie referral hospital [16–18].

It is well known that IOL plays a role in reducing maternal morbidity and mortality associated with pregnancy and pregnancy-related complications. However, IOL is not always successful, sometimes it fails and necessitate emergency CS delivery which is reported to have many adverse maternal and neonatal health outcomes including post-partum hemorrhage (PPH), hysterectomy, wound complications, sepsis [14], neonatal injuries, maternal death [7, 19, 20], and longer recovery period [17, 21, 22]. The risk of CS delivery and other operative deliveries are higher among women with induced labor than women with spontaneous onset of labor [7, 23–25]. A study conducted at Mattu Karl Hospital, Ethiopia reported that 35.5% of neonates and 6.5% of women had adverse outcomes as a result of FIOL [26].

Advanced maternal age, nulliparity, poor Bishop Score, pregnancy-induced hypertension (PIH), premature rupture of membrane (PROM), and post-term pregnancy are among the contributing factors for FIOL. Nevertheless, there are inconsistencies regarding these factors.

Despite the steady increase in the failure rate of induction [17, 18], a little was known on the prevalence and factors associated with FIOL in Ethiopia. Particularly in the study area, there was a paucity of information on the prevalence and factors associated with FIOL. Therefore, this study aimed to fill the gap in lack of sufficient evidence in the prevalence of failed induction of labor and its associated factors among women undergoing induction at the University of Gondar Specialized Hospital, Northwest Ethiopia.

## Methods

### Study area and period

The study was conducted at the University of Gondar Specialized Hospital (UoGSH), Northwest Ethiopia, from March 1 to 30, 2020, where Gondar is its capital city located 727 km northwest of Addis Ababa, the capital city of Ethiopia. The Hospital provides referral and primary maternal health services for an estimated population of more than 5 million. Currently, the hospital holds 550 beds, of which 58 beds serve for obstetric admissions [27]. As the information obtained from the clinician working there, a minimum of three and a maximum of seven pregnant women were admitted to the labor ward for IOL. The prevalence of cesarean section in the study setting was 29.7% (Unpublished) [28].

### Study design

We employed an institutional-based retrospective cross-sectional study.

### Participants and sampling procedure

All women undergoing IOL at UoGSH were the source population whereas women undergoing IOL handled from the 1st January 2018 to 31st December 2019 at UoGSH were the study population. All medical cards of women who experienced IOL with at least a medical history sheet or the induction sheet attached in their medical card were included in the study irrespective of parity status. However, medical registration numbers (MRN) of women undergoing IOL that could not be read from the maternity registration books were excluded from the study. The sample size was calculated by using a single population proportion formula; $\mathrm{n}=\frac{{\left(\mathrm{Z}\upalpha /2\right)}^2\mathrm{p}\left(1-\mathrm{p}\right)}{{\mathrm{d}}^2}=\frac{\ {(1.96)}^{2\ast }0.197\left(1-0.197\right)}{(0.03)\ 2}=675.$

where “n” is the total sample size, “p” is the proportion of failed induction of labor taken from a study conducted in Dessie referral hospital which was 19.7% [16], “d” is a 3% margin of error, “α” is taken at 95% level of significance. Adding a 10% non-response rate (10% for inaccessible cards), the final sample size of 743 has been determined.

The sampling procedure was done from the maternity registration books; first, all medical registration numbers (MRN) of women who experienced IOL and registered from 1st January 2018 to 31st December 2019 were picked from the maternity registration books which was 2026(N). Then, the sampling frame was prepared by numbering those MRN from 1 to N. Skipping interval (k^th^ interval) was computed by dividing the total population (N) who undergone IOL within the 2 years period for the total sample size (n). Finally, the total sample size was selected by systematic random sampling method.

### Data collection tools and procedure

Data extraction form adapted from different published literature [11, 17, 18, 21, 29] was used to extract data on the socio-demographic, obstetric, reason for induction, method, and dose-related factors. The maternity registration books, the client’s card with medical history sheet, induction sheet, labor follow-up sheet, partograph sheet, and operation note sheet were assessed to extract the required data. BSc and MSc midwives were recruited for the data extraction process since they are closer to the topic area so that they could extract data easily and accurately. To assure the quality of data, two-day intensive training was given for all the data collectors and supervisors on the overall process of the data collection procedure.

### Operational definitions

- **Failed induction of labor** (FIOL): is operationalized as the occurrence of birth by CS for the indication of failed induction of labor [10–12, 17, 30].
- **Bishop Score**: scored out of 13 points using five parameters and used to assess cervical favorability status [12, 31].
- **Artificial rupture of membrane**: when a doctor or midwife puts a small hole in the bag of the membranes or waters around the baby [32].
- **Premature rupture of membrane**: rupture of the membrane before the onset of labor [33, 34].
- **Pregnancy-induced hypertension**: maternal hypertension of any type with a blood pressure of ≥140/90 mmHg during pregnancy and childbirth [12].
- **Nulliparous**: a woman whose pregnancy never carried to viability.

### Data processing and analysis

The data extracted from the clinical record were checked manually for completeness, and then cleaned and coded. Then, data were exported to SPSS version 20 for analysis. We performed descriptive statistics to characterize the study population. Frequency tables, pie-chart, and graphs were used to display the results. Bivariable and multivariable logistic regression models were done to identify factors associated with FIOL. Variables with a *p*-value *p* < 0.25 in the bivariate analysis were entered into a multivariable logistic regression model for controlling possible confounders. Finally, the odds ratio with a *p*-value *p* < 0.05 in the multivariable logistic regression model was considered statistically significant. Multicollinearity was checked by using variance inflation factor(VIF), VIF > 10 was considered as multicolliner. Model fitness was assessed by the Hosmer-Lemeshow test which was 0.8.

### Induction methods

The induction procedure performed in Ethiopia varies from institution to institution. The dose and methods of induction vary across the institutions. For example, 5 IU for primigravida and 2.5 IU for multigravida is the recommended initial dose in our study setting. However in other institutions, the dose may be similar irrespective of gravida status. The preferred method of induction could be selected based on the indications or the health care provider’s decision. But, the most commonly used method of induction in our setting is oxytocin infusion and the most commonly used method of cervical ripening is a Balloon catheter.

To start induction, the clinicians first assessed cervical favorability or maturity by using the Bishop Score. If the Bishop Score is less than 5, induction may not be initiated, cervical ripening may be needed or the induction may be postponed depending on the indications [9]. The cervix is considered to be matured if the Bishop Score is 6 or more.

### Bishop scoring

**Table:** 

Score	Dilation (cm)	Effacement (%)	Station (cm)	Consistency	Position
0	Closed	0–30	−3	firm	Posterior
1	1 − 2 cm	40–50	-2	Medium	Mid position
2	3-4 cm	60–70	−1,0	Soft	Anterior
3	≥5 cm	≥80	+ 1,+ 2		

## Results

### Socio-demographic characteristics

A total of 713 medical records of women who had undergone IOL were included in the study, 30 medical records were not accessed during the data collection time for different reasons, making an accessibility rate of almost 96%. The mean age ± SD of the women was 27.87 ± 3.66, and more than three-quarters (76.3%) of the study participants were younger than 30 years. Regarding women’s place of residence, 457(64.4%) were urban residents (Table 1).

**Table 1:** Socio-demographic characteristics of women undergoing induction at University of Gondar Specialized Hospital, Northwest Ethiopia, 2020

Variables	Frequency	%
**Age in years** (*n* = 713)		
≤ 30	544	76.3
> 30	169	23.7
**Place of residence**(*n* = 710)		
Urban	457	64.4
Rural	253	35.6
Residence not recorded (missing = 3)		

### Obstetric related characteristics

Among 713 induced women, 520 (72.9%) were nulliparous. From 663 women who had a known last normal menstrual period(LNMP), the mean gestational age(GA) ± SD was 39 ± 2 weeks and 263(39.7%) of them were between 38 and 40 weeks of gestation. Of 698 induced women, 499 (71.5%) had a newborn with a birth weight of 2500-3999 g. Out of 691 induced women, 370(53.5%) had a bishop score of greater than 5. Among 678 women, 644 (95%) have stayed for less than or equal to 18 h duration in the latent first stage of labor (Table 2).

**Table 2:** Obstetric related characteristics of women undergoing induction at University of Gondar Specialized Hospital, Northwest Ethiopia, 2020

Variables	Frequency	%
**Parity status at the time of induction** (*n* = 713)		
Nulliparous	520	72.9
Multiparous	193	27.1
**Birth weight of the newborn in gram in the current pregnancy** (*n* = 698)		
< 2500 g	157	22.5
2500-3999 g	499	71.5
≥ 4000 g	42	6.0
Birth weight not recorded (missing = 15)		
**Gestational age in weeks at times of induction** (*n* = 663)		
≤ 37	252	38.0
38–40	263	39.7
≥ 41	148	22.3
Unknown LNMP (missing = 50)		
**Duration of the latent first stage of labor in current labor** (*n* = 678)		
≤ 18 h	644	95.0
> 18 h	34	5.0
Duration not recorded (missing = 35)		
**Pre-induction bishop score in the current labor** (*n* = 691)		
≤ 5	321	46.5
> 5	370	53.5
Bishop score not recorded (missing = 22)		

### Reasons for induction related characteristics

**Fig. 1:**
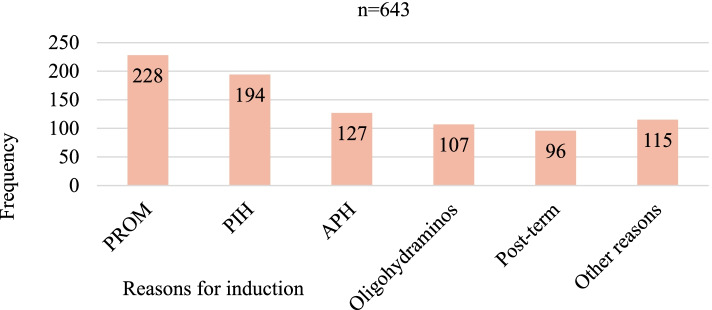
Reasons for induction of labor among women undergoing induction at University of Gondar Specialized Hospital, Northwest Ethiopia, 2020. Other reasons: Intra-uterine growth restriction, Diabetic Mellitus, and Polyhydramnios. Key: PROM: Premature rupture of membrane, PIH: pregnancy-induced hypertension, APH: antepartum hemorrhage

Among the total, a clear reason for the initiation of induction was documented in 643(90.2%) of the participant; of these, PROM was the predominant indication accounted 228(35.5%) followed by PIH, 194 (30.2%) (Fig. 1).

### Method and dose-related characteristics

**Fig. 2:**
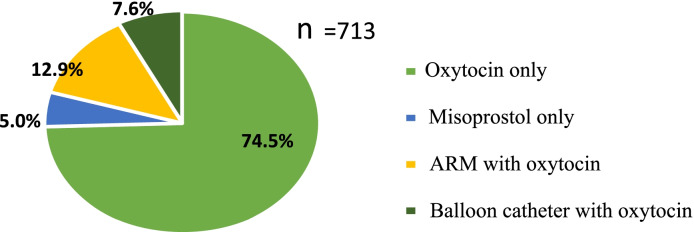
The different induction methods among women undergoing induction at University of Gondar Specialized Hospital, Northwest Ethiopia, 2020. Key: ARM: artificial rupture of membrane, IU: International Unit

Among the total, 531(74.5%) of them were induced only with oxytocin infusion followed by a combination of ARM with oxytocin infusion 92(12.9%) (Fig. 2). Among women induced only with oxytocin infusion, 346(65.2%) were induced with a maximum dose of ≤5 IU, 158(29.8%) 6 to 10 IU, and 27(5%) greater than 10 IU.

### Prevalence of failed induction of labor

#### Modes of delivery

**Fig. 3:**
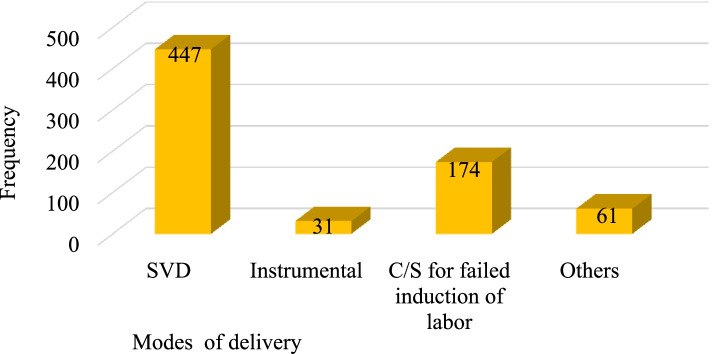
Modes of delivery among women undergoing induction at University of Gondar Specialized Hospital, Northwest Ethiopia, 2020. Others; CS for fetal distress, severe pre-eclampsia/eclampsia, severe vaginal bleeding, failed instrumental delivery, and Cephalo-pelvic disproportion (CPD)

Among the total induced women, 447 (62.7%) gave birth through spontaneous vertex delivery (SVD) followed by 174(24.4%) by cesarean section (CS) for failed induction of labor (Fig. 3).

#### Factors associated with failed induction of labor

In bivariate analysis; age, place of residence, parity, birth weight, pre-induction bishop score, post-term pregnancy, premature rupture of membrane (PROM), pregnancy-induced hypertension (PIH), and method of induction were significantly associated with FIOL. However, in the multivariable analysis, age, place of residence, parity, bishop score, PROM, PIH, and method of induction showed a significant association with FIOL. Women older than 30 years were 3.7 times more likely to have FIOL than women with age less than or equal to 30 years. Women from the rural area were 3.7 times more likely to have FIOL than women from urban. The odds of FIOL were 2.1 times more likely among nulliparous women compared to multiparous women. Women with pre-induction bishop scores of less than or equal to 5 were 3.4 times more likely to have FIOL than those with a bishop score of greater than 5. The odds of FIOL were 2.7 times more likely among women with PROM than women without it. Women with PIH were 4 times more likely to have FIOL compared to women without PIH. The odds of FIOL were 80% times less likely among women who were induced with combinations of ARM with oxytocin as compared to women induced only with oxytocin infusion (Table 3).

**Table 3:** Bivariable and multivariable logistic regression analysis of factors associated with failed induction of labor among women undergoing induction at University of Gondar Specialized Hospital, Northwest Ethiopia, 2020

Variables	Failed induction of labor		95% Confidence Interval	
Name and Label	Yes	No	COR	AOR
**Age in years**				
≤ 30	105(19.3%)	439(80.7%)	1	1
> 30	69(40.8%)	100(59.2%)	2.9(2.0,4.2)***	3.7(2.2,6.2)***
**Residence**				
Urban	65(14.2%)	392(85.8%)	1	1
Rural	108(42.7%)	145(57.3%)	4.5(3.1,6.5)***	3.7(2.4,5.8)***
**Parity status at time of induction**				
Nulliparous	139(26.7%)	381(73.3%)	1.6(1.1,2.5)*	2.1(1.2,3.7)**
Multiparous	35(18.1%)	158(81.9%)	1	1
**Birth weight in the current pregnancy**				
< 2500	54(34.4%)	103(65.6%)	2.0(1.4,2.9)***	1.5(0.9,2.5)
2500–3999	103(20.6%)	396(79.4%)	1	1
≥ 4000	15(35.7%)	27(64.3%)	2.1(1.1,4.2)*	1.7(0.7,4.2)
**Gestational age at the time of induction**				
≤ 37	69(27.4%)	183(72.6%)	1	
38–40	55(20.9%)	208(79.1%)	0.7(0.5,1.1)	
≥ 41	28(18.9%)	120(81.1%)	0.6(0.4,1.0)	
**Pre-induction bishop score**				
≤ 5	120(37.4%)	201(62.6%)	4.3(2.9,6.3)***	3.4(2.2,5.4)***
> 5	45(12.2%)	325(87.8%)	1	1
**Duration of the latent phase**				
≤ 18 h	155(24.1%)	489(75.9%)	1	
> 18 h	10(29.4%)	24(70.6%)	1.3(0.6,2.8)	
**Post-term pregnancy**				
No	149(27.2%)	398(72.8%)	1	1
Yes	15(15.6%)	81(84.4%)	0.5(0.3,0.9)*	1.4(0.7,3.0)
**Premature rupture of membrane**				
No	93(22.4%)	322(77.6%)	1	1
Yes	71(31.1%)	157(68.9%)	1.6(1.1,2.3)*	2.7(1.5,4.6)***
**Intra-uterine growth restriction**				
No	153(25.7%)	442(74.3%)	1	
Yes	11(22.9%)	37(77.1%)	0.9(0.4,1.7)	
**Pregnancy-induced hypertension**				
No	90(20%)	359(80%)	1	1
Yes	74(38.1%)	120(61.9%)	2.5(1.7,3.6)***	4.0(2.3,7.1)***
**Diabetic Mellitus**				
No	152(24.9%)	458(75.1%)	1	
Yes	12(36.4%)	21(63.6%)	1.7(0.8,3.6)	
**Antepartum hemorrhage**				
No	132(25.6%)	384(74.4)	1	
Yes	32(25.2%)	95(74.8%)	1.0(0.6,1.5)	
**Oligohydramnios**				
No	136(25.4%)	400(74.6%)	1	
Yes	28(26.2%)	79(73.8%)	1.0(0.7,1.7)	
**Polyhydramnios**				
No	153(25.1%)	456(74.9%)	1	
Yes	11(32.4%)	23(67.6%)	1.4(0.7,2.9)	
**Method of induction**				
Oxytocin infusion only	138(26%)	393(74%)	1	1
Misoprostol only	11(30.6%)	25(69.4%)	1.3(0.6,2.6)	
ARM with oxytocin	14(15.2%)	78(84.8%)	0.5(0.3,0.9)*	0.2(0.1,0.4)***
Balloon catheter with oxytocin	11(20.4%)	43(79.6%)	0.7(0.4,1.5)	
**Maximum oxytocin dose used**				
≤ 5 IU	93(26.9%)	253(73.1%)	1	
6-10 IU	33(20.9%)	125(79.1%)	0.7(0.5,1.1)	
> 10 IU	12(44.4%)	15(55.6%)	2.2(0.9,4.8)	

*AOR:* Adjusted Odds Ratio, *COR* Crude Odds Ratio, 1: Reference category

^*^Significant at *p* < 0.25 but > 0.01

^**^Significant at *P* = 0.01

^***^Significant at *P* = 0.000

## Discussion

This study showed that the prevalence of failed induction of labor in the study area is 24.4% which is comparable with studies conducted in Jimma University Specialized Hospital (21.4%) [17], Dessie referral hospital, Ethiopia (19.7%) [16], and Hawassa public health facilities, Ethiopia (17.3%) [18]. However, the study finding showed a higher prevalence of FIOL than a study conducted in Seongnamsi, Korea (14%) [35]. The reason might be due to the difference in the selection of parity status in which all parity status included in this study but only multipara women were included in Korea. Because the evidence showed that the likelihood of FIOL is less among multiparous women.

On the other hand, the prevalence of FIOL in this study is lower than studies conducted in Adelaide, Australia (42%) [10] and Trabzon, Turkey (35.2%) [36]. The discrepancy might be due to the difference in the study setting in which there might be well-equipped CS facilities in developed countries which would increase the likelihood of CS delivery for FIOL. Variations in the commonly used methods of induction such as oxytocin is common in the study area whereas misoprostol is common in some other settings might also be the reason for the difference.

This study showed that as the maternal age increases, the odds of FIOL increase which might be because advanced maternal age puts them at greater risk of complications like PIH and DM. This is supported by previous studies done in Hawassa, Ethiopia [18], Nepal [29], Australia [10], and Mansoura University Hospital, and Dikirnis Hospital [37]. This may further explain the fact that as age increases, myometrial contractility decreases which can result in poor uterine contraction and the subsequently, FIOL [37, 38].

The study showed a significant association between residence and FIOL, the odds of FIOL were more likely among rural resident women than urban residents. The possible reasons for this might be women from the rural area may not come for ANC follow-up for early detection of pregnancy-related complications like PIH and PROM or may not totally come to health institutions or they may come too late after complicated pregnancy which can lead to FIOL. The finding is consistent with a study conducted in Dessie referral hospital, Ethiopia [16].

This study showed the likelihood of FIOL increased among nulliparous women compared to multiparous women. This may partly be due to the fact that nulliparous women are different from multiparous women in pre-induction cervical status as well as response to induction methods [2, 12, 39]. In addition, the nulliparous’ cervix is immature and requires a longer time and effort to stimulate through induction compared with multiparous women. One study conducted in Eastern Ethiopia explained that, as parity increases, the likelihood of FIOL decreases because uterine muscles can be easily stimulated and contracted in multipara women [40]. Moreover, this finding was consistent with the findings studies conducted in Sungailiat regional public hospital, Bangka district [39], Hawassa [18], Pakistan [11], Nepal [29], and Saudi Arabia [41].

FIOL increased in women with low pre-induction Bishop Score (BS), this might be because of the fact that cervical status is a fundamental parameter in BS. In low BS, the cervix is in an unfavorable state which can increase the likelihood of FIOL [42–44]. The finding is consistent with previous findings conducted in Hawassa [18], Jimma [17], Pakistan [11], and India [45]. However, this finding contradicts the finding of a study conducted in Dessie referral hospital, Ethiopia which showed that FIOL was less likely among women with poor Bishop Score [16]. This contrast might be related to the subjective assessment of the BS. The smaller sample size included in a study at Dessie referral hospital might also be an additional reason for this discrepancy. Nevertheless, most previous findings well documented that the odds of FIOL were more likely among women with poor BS and this is supported by the study finding [11, 17, 18, 45].

Furthermore, the odds of FIOL increases in women with PROM being an indication of induction of labor. This might be because of the fact that PROM can affect the time given for cervical ripening or labor induction, sufficient time may not be given to ripen the cervix or to achieve the active phase of labor due to fear of infection. An additional explanation might be, fear of using cervical ripening methods specifically inserting mechanical methods to ripen the cervix because of fear of infection. This finding is comparable with studies conducted in Ethiopia [18] and Pakistan [11].

In this study, PIH was strongly associated with FIOL. The possible explanation might be the fact the drug Magnesium sulfate (MgSo4) which is given for the management of PIH (pre-eclampsia /eclampsia) is a known tocolytic drug [46, 47] that can arrest labor and result in poor progress of labor and FIOL [48]. The finding is consistent with studies conducted in Mayo Clinic in Rochester, Ferrara University, and University of Washington Medical Center supported this finding [49–51].

Lastly, FIOL was 80% times less in the induction regimen with a combination of ARM with oxytocin infusion. This might be due to the fact ARM initiates the release of endogens prostaglandin and increases the strength of uterine contraction, which might result in successful IOL. The use of a combination method for successful induction of labor was suggested in previous findings [23, 31, 37, 52].

## Conclusion

In this study, 24.4% of women who underwent induction experienced failed induction of labor. Older maternal age, being rural residence, nulliparity, less than 5 Bishop score, PROM, and PIH were among the variables, which increased the likelihood of FIOL. On the other hand, using combinations of ARM with oxytocin as a method of labor induction decreased the likelihood of FIOL. A combination method of ARM with oxytocin is highly recommended for successful induction. Those women mainly who are nulliparous and have poor bishop scores should ripen the cervix before initiation of induction by using different cervical ripening methods. Early detection and treatment of pregnancy-related complications like PIH and PROM are also recommended. Researchers are recommended to do further randomized control trials (RCT), to have a better understanding of the effective induction methods.

## Supplementary Information

**Additional file 1: S1 Table.** SPSS data for failed induction of labor and associated factors.

## Data Availability

The dataset used/analyzed during the current study are available and included in the supplementary information files.

## Abbreviations

ARM: Artificial Rupture of Membrane
CS: Cesarean Section
FIOL: Failed Induction of Labor
IOL: Induction of Labor
IU: International Unit
PIH: Pregnancy Induced Hypertension
PROM: Premature Rupture of Membrane
UoGSH: University of Gondar Specialized Hospital
